# Effect of Music Therapy on Anxiety in Pregnancy: A Systematic Review of Randomized Controlled Trials

**DOI:** 10.7759/cureus.69066

**Published:** 2024-09-10

**Authors:** Naseema Shafqat, Amit Agrawal, K Pushpalatha, Bharti Singh, Ranjana Verma, Lily Podder, Saikat Das, Roshan F Sutar

**Affiliations:** 1 Obstetrics and Gynecology Nursing, All India Institute of Medical Sciences, Bhopal, Bhopal, IND; 2 Neurosurgery, All India Institute of Medical Sciences, Bhopal, Bhopal, IND; 3 Obstetrics and Gynecology, All India Institute of Medical Sciences, Bhopal, Bhopal, IND; 4 Medical Surgical Nursing, All India Institute of Medical Sciences, Bhopal, Bhopal, IND; 5 Radiotherapy, All India Institute of Medical Sciences, Bhopal, Bhopal, IND; 6 Psychiatry, All India Institute of Medical Sciences, Bhopal, Bhopal, IND

**Keywords:** anxiety, maternal well-being, meta-analysis, music therapy, pregnancy, prenatal anxiety, randomized controlled trials, systematic review

## Abstract

Pregnancy can heighten anxiety levels, impacting both maternal and fetal well-being. This systematic review synthesizes evidence from 33 randomized controlled trials exploring the effects of music therapy on anxiety in pregnant women. The studies included diverse populations, ranging from women undergoing in vitro fertilization (IVF) to those facing high-risk pregnancies. Music therapy interventions varied widely, including virtual reality experiences, classical music, lullabies, and patient-selected music, administered at different pregnancy stages such as IVF treatments, elective cesarean sections, and high-risk hospitalizations. The findings consistently demonstrated that music therapy significantly reduces anxiety levels in pregnant women. Positive outcomes included reductions in both state and trait anxiety, improved pregnancy rates, and enhanced maternal-fetal parameters. Additionally, music therapy showed promise in reducing anxiety during labor, cesarean deliveries, and high-risk hospital stays. These varied interventions and their positive outcomes highlight the potential of music therapy as an effective, non-pharmacological approach to managing pregnancy-related anxiety. This review provides a comprehensive overview of the existing evidence on music therapy's efficacy in alleviating anxiety during pregnancy. It underscores the need for further research to standardize interventions and incorporate music therapy into routine prenatal care. By enhancing the overall well-being of expectant mothers, music therapy could become a valuable adjunct to conventional prenatal care practices.

## Introduction and background

Pregnancy, while often a joyful period, can also bring significant anxiety for expectant mothers [1]. High levels of anxiety can lead to increased stress hormones like cortisol, which may affect the developing baby's growth and development. Additionally, it can contribute to complications such as preterm birth and low birth weight [2]. The negative, enduring, persistent, and sometimes irreversible effects of anxiety during pregnancy can turn this experience into a distressing and unpleasant phase in a woman's life. It can adversely impact both maternal and fetal well-being if not managed effectively. Therefore, expectant mothers need to seek support from healthcare providers, practice relaxation techniques, maintain a healthy lifestyle, and communicate openly about their concerns [1,2]. Seeking counseling or joining support groups can also be beneficial in managing anxiety during pregnancy. Traditional approaches to managing antenatal anxiety frequently involve pharmacological interventions, which, despite their efficacy, come with potential side effects and risks that can be concerning for both the mother and the developing fetus. This has led to a growing interest in non-pharmacological, holistic methods that align with the principles of patient-centered care. One such promising intervention is music therapy.

Music therapy is a therapeutic approach that uses music to address various physical, emotional, cognitive, and social needs. It is an evidence-based practice conducted by credentialed professionals who have completed approved music therapy programs. The use of music therapy in healthcare is not a new concept. It has been used for decades to improve symptoms of various conditions, including dementia, depression, and pain. The American Music Therapy Association defines music therapy as the clinical and evidence-based use of music interventions to accomplish individualized goals within a therapeutic relationship by a credentialed professional [3]. Music therapy can include active techniques, such as improvisation, singing, clapping, or dancing, and receptive techniques, where the client listens to music to identify its emotional content. Music's versatility and universal appeal make it a unique therapeutic tool that transcends language and cultural barriers, offering a non-invasive avenue for emotional expression and support [4,5].

A growing body of evidence suggests that music therapy can effectively reduce anxiety levels. Studies have shown that music therapy can improve mood, reduce stress, and promote relaxation. In the realm of obstetrics, research has indicated that music therapy can alleviate anxiety during labor and delivery, reduce the perception of pain, and enhance the overall birthing experience [6,7].

The theoretical framework underlying the use of music therapy for anxiety reduction is based on several mechanisms. Music has been found to activate neural pathways associated with reward, emotion, and arousal regulation [8]. Listening to music can release neurotransmitters such as dopamine and endorphins, which promote feelings of pleasure and well-being [9]. Additionally, music can modulate physiological responses by reducing cortisol levels, lowering heart rate, and decreasing blood pressure, all of which are markers of reduced stress and anxiety [10].

In the context of pregnancy, anxiety can arise from various sources, including concerns about the health of the baby, the labor and delivery process, and the significant life changes that come with parenthood. Elevated anxiety levels during pregnancy are associated with adverse outcomes such as preterm birth, low birth weight, and postpartum depression. Therefore, finding effective ways to manage anxiety during this period is crucial. The potential of music therapy to alleviate anxiety has been recognized in various medical fields, including obstetrics. Its application in pregnancy is particularly appealing as it provides a safe, non-pharmacological alternative for managing anxiety, which can be seamlessly integrated into routine prenatal care [11,12]. This systematic review aims to critically examine the existing literature on the impact of music therapy on anxiety levels in pregnant women. The objectives are twofold: first, to measure and analyze the reduction in anxiety levels among pregnant women following music therapy interventions; and second, to investigate any adverse effects or unintended consequences associated with music therapy interventions in this population.

Methods

A comprehensive systematic review protocol was meticulously developed and registered at PROSPERO, outlining research questions, search strategy, inclusion criteria, and data extraction and analysis methods. The protocol adheres to established guidelines, including the Preferred Reporting Items for Systematic Reviews and Meta-Analyses (PRISMA) guidelines and the Cochrane Manual of Systematic Reviews and Meta-Analyses, ensuring transparency and minimizing bias.

Eligibility criteria

The systematic review utilized predetermined criteria based on the Population, Intervention, Comparison, and Outcome (PICO) framework to guide study selection. Inclusion criteria encompassed randomized controlled trials (RCTs) involving pregnant women, investigating the impact of music therapy interventions on anxiety levels. Exclusion criteria excluded studies outside the defined scope, those lacking the necessary outcome measures, non-English publications and non-RCTs, quasi-experimental studies, prospective and retrospective observational studies, case series, case reports, letters, editorials, comments, and animal studies (Table 1).

**Table 1:** Inclusion or exclusion criteria Population, Intervention, Comparison, and Outcome (PICO) criteria used in the selection of studies

Parameter	Inclusion Criteria	Exclusion Criteria
Type of study design	Randomized controlled trials (RCTs): Designed to compare the effect of music therapy for pregnancy-related anxiety to a control group.	Non-interventional studies: Observational studies assessing the effect of music therapy without intervention. Systematic reviews and meta-analyses: Review articles that provide relevant data or studies included in meta-analyses. Qualitative studies: Studies not providing quantitative data on the effect of music therapy. Case reports: Individual patient cases without group-level data.
Population	Pregnant women: Studies involving pregnant women of any age, gestational stage, and cultural background.	Non-pregnant individuals: Studies involving populations unrelated to pregnancy. Non-human studies: Animal or laboratory studies not directly applicable to pregnant women.
Intervention	RCTs on music therapy, which includes various musical techniques, such as listening to music, singing, or playing musical instruments, with the aim of reducing anxiety levels during pregnancy.	Interventions other than music therapy.
Control	Studies with a control group or comparator for evaluating the effect of music therapy on pregnancy-related anxiety. Studies with adequate control measures, including standard clinical assessments or alternative interventions.	Studies without a control group for comparison. Studies with inadequate control measures that do not allow for the assessment of diagnostic accuracy.
Outcome	Studies reporting outcomes for music therapy in the context of pregnancy-related anxiety. Studies providing sufficient data.	Studies without relevant outcomes. Studies without adequate data.

Search strategy

Using predefined search terms, a systematic search was conducted across multiple electronic databases, including PUBMED, SCOPUS, the Central Cochrane Registry of Controlled Trials (the Cochrane Library), and ScienceDirect. The search covered the period from inception to December 31, 2023, independently conducted by multiple reviewers with regular meetings to resolve discrepancies and involving a third reviewer in case of disagreements (Table 2).

**Table 2:** Details of search strategy

Database	Search Terms
PubMed	(("music"[MeSH Terms] OR "music"[All Fields] OR "music s"[All Fields] OR "musical"[All Fields] OR "musicality"[All Fields] OR "musically"[All Fields] OR "musicals"[All Fields] OR "musics"[All Fields]) AND ("pregnancy"[MeSH Terms] OR "pregnancy"[All Fields] OR "pregnancies"[All Fields] OR "pregnancy s"[All Fields]) AND ("anxiety"[MeSH Terms] OR "anxiety"[All Fields] OR "anxieties"[All Fields] OR "anxiety s"[All Fields])) AND ((randomizedcontrolledtrial[Filter]) AND (1000/1/1:2023/10/13[pdat]))
SCOPUS	TITLE-ABS-KEY music pregnancy anxiety
COCHRANE	7 Cochrane Reviews matching music pregnancy anxiety in Title Abstract Keyword
ScienceDirect	Title, abstract, keywords: music pregnancy anxiety

## Review

Process details

Screening/Selection Process

Two independent reviewers (NS and AA) screened each record against inclusion criteria, with any disagreements resolved through consensus discussions or the involvement of a third reviewer (RV). This ensured reliability and minimized bias in the screening process.

Data Extraction Process

Data extraction adhered to a systematic and transparent approach. Two reviewers (NS and AA) independently evaluated and extracted data from each study using a predefined proforma. Periodic reviews and contact with authors ensured accuracy, with discrepancies resolved through consensus discussions and the involvement of a third party when necessary.

Data Items

Outcomes, including anxiety levels, participant characteristics, and intervention details, were meticulously identified and defined. The systematic review sought to capture a holistic view of relevant outcomes and variables while maintaining transparency in decision-making processes.

Study Risk of Bias Assessment

The Joanna Briggs Institute (JBI) tool was employed to assess the risk of bias within the included studies [13]. Two reviewers (NS and AA) independently assessed studies, following JBI guidelines, with consensus discussions and periodic reviews resolving discrepancies or disagreements (Table 3).

**Table 3:** Risk of bias assessment [14-46]

Study/Author	Item 1	Item 2	Item 3	Item 4	Item 5	Item 6	Item 7	Item 8	Item 9	Item 10	Item 11	Item 12	Item 13
Almedhesh et al., 2022 [14]	Yes	Yes	Yes	Yes	Unclear	Yes	Yes	Yes	Yes	Yes	Yes	Yes	Yes
Baltaci et al., 2022 [15]	Yes	Yes	Yes	No	No	Yes	No	Yes	Yes	Yes	Yes	Yes	Yes
Buglione et al., 2020 [16]	Yes	Yes	Yes	Yes	Unclear	Yes	Yes	Yes	Yes	Yes	Yes	Yes	Yes
Cao et al., 2016 [17]	Yes	Unclear	Yes	Unclear	Unclear	Yes	Unclear	Yes	Yes	Yes	Yes	Yes	Yes
Catalgol et al., 2022 [18]	Yes	Yes	Yes	Yes	Yes	Yes	Yes	Yes	Yes	Yes	Yes	Yes	Yes
Chang et al., 2005 [19]	Yes	Yes	Yes	Unclear	Unclear	Yes	Unclear	Yes	Yes	Yes	Yes	Yes	Yes
Chang et al., 2008 [20]	Yes	No	Yes	Unclear	Unclear	Yes	Unclear	Yes	Yes	Yes	Yes	Yes	Yes
Drzymalski et al., 2023 [21]	Yes	Yes	Yes	No	No	Yes	No	Yes	Yes	Yes	Yes	Yes	Yes
Drzymalski et al., 2017 [22]	Yes	Yes	Yes	Yes	Unclear	Yes	Unclear	Yes	Yes	Yes	Yes	Yes	Yes
Drzymalski et al., 2020 [23]	Yes	Yes	Yes	Yes	Unclear	Yes	Unclear	Yes	Yes	Yes	Yes	Yes	Yes
Estrella-Juarez et al., 2023 [24]	Yes	Yes	Yes	Yes	Yes	Yes	Yes	Yes	Yes	Yes	Yes	Yes	Yes
Garcia-Gonzalez et al., 2018 [25]	Yes	Yes	Yes	Yes	Unclear	Yes	Unclear	Yes	Yes	Yes	Yes	Yes	Yes
Gokyildiz Surucu et al., 2018 [26]	Yes	Yes	Yes	No	No	Yes	No	Yes	Yes	Yes	Yes	Yes	Yes
Handan et al., 2018 [27]	No	No	Yes	No	No	Yes	No	Yes	Yes	Yes	Yes	Yes	Yes
Hanprasertpong et al., 2016 [28]	Yes	Yes	Yes	Yes	Yes	Yes	Yes	Yes	Yes	Yes	Yes	Yes	Yes
Hepp et al., 2018 [29]	Yes	Unclear	Yes	Unclear	Unclear	Yes	Unclear	Yes	Yes	Yes	Yes	Yes	Yes
Kakde et al., 2023 [30]	Yes	Yes	Yes	Yes	Unclear	Yes	Unclear	Yes	Yes	Yes	Yes	Yes	Yes
Li et al., 2012 [31]	Yes	Unclear	Yes	Unclear	Unclear	Yes	Unclear	Yes	Yes	Yes	Yes	Yes	Yes
Liu et al., 2010 [32]	Yes	Unclear	Yes	Unclear	Unclear	Yes	Unclear	Yes	Yes	Yes	Yes	Yes	Yes
Liu, et al., 2016 [33]	Yes	Yes	Yes	Yes	Unclear	Yes	Unclear	Yes	Yes	Yes	Yes	Yes	Yes
Momeni et al., 2020 [34]	No	Yes	Yes	No	No	Yes	No	Yes	Yes	Yes	Yes	Yes	Yes
Nwebube et al., 2017 [35]	Yes	Yes	Yes	Yes	Yes	Yes	Unclear	Yes	Yes	Yes	Yes	Yes	Yes
Parodi et al., 2021 [36]	Yes	Yes	Yes	Yes	Yes	Yes	Yes	Yes	Yes	Yes	Yes	Yes	Yes
Reza et al., 2007 [37]	Yes	Yes	Yes	Yes	Yes	Yes	Yes	Yes	Yes	Yes	Yes	Yes	Yes
Rezaei et al., 2023 [38]	Yes	Unclear	Yes	Unclear	Unclear	Yes	Unclear	Yes	Yes	Yes	Yes	Yes	Yes
Simavli et al., 2014 [39]	Yes	Yes	Yes	Yes	Yes	Yes	Yes	Yes	Yes	Yes	Yes	Yes	Yes
Simavli et al., 2014 [40]	Yes	Yes	Yes	Yes	Yes	Yes	Yes	Yes	Yes	Yes	Yes	Yes	Yes
Soylu et al., 2022 [41]	Yes	No	Yes	No	No	Yes	No	Yes	Yes	Yes	Yes	Yes	Yes
Teckenberg-Jansson et al., 2019 [42]	Yes	Yes	Yes	Yes	No	Yes	Yes	Yes	Yes	Yes	Yes	Yes	Yes
Toker et al., 2017 [43]	Yes	Yes	Yes	Yes	Unclear	Yes	Unclear	Yes	Yes	Yes	Yes	Yes	Yes
Ventura et al., 2012 [44]	Yes	Yes	Yes	Unclear	Unclear	Yes	Unclear	Yes	Yes	Yes	Yes	Yes	Yes
Wu et al., 2012 [45]	Yes	Yes	Yes	No	No	Yes	Yes	Yes	Yes	Yes	Yes	Yes	Yes
Yüksekol et al., 2020 [46]	Yes	Yes	Yes	Yes	No	Yes	No	Yes	Yes	Yes	Yes	Yes	Yes

Results

**Figure 1:**
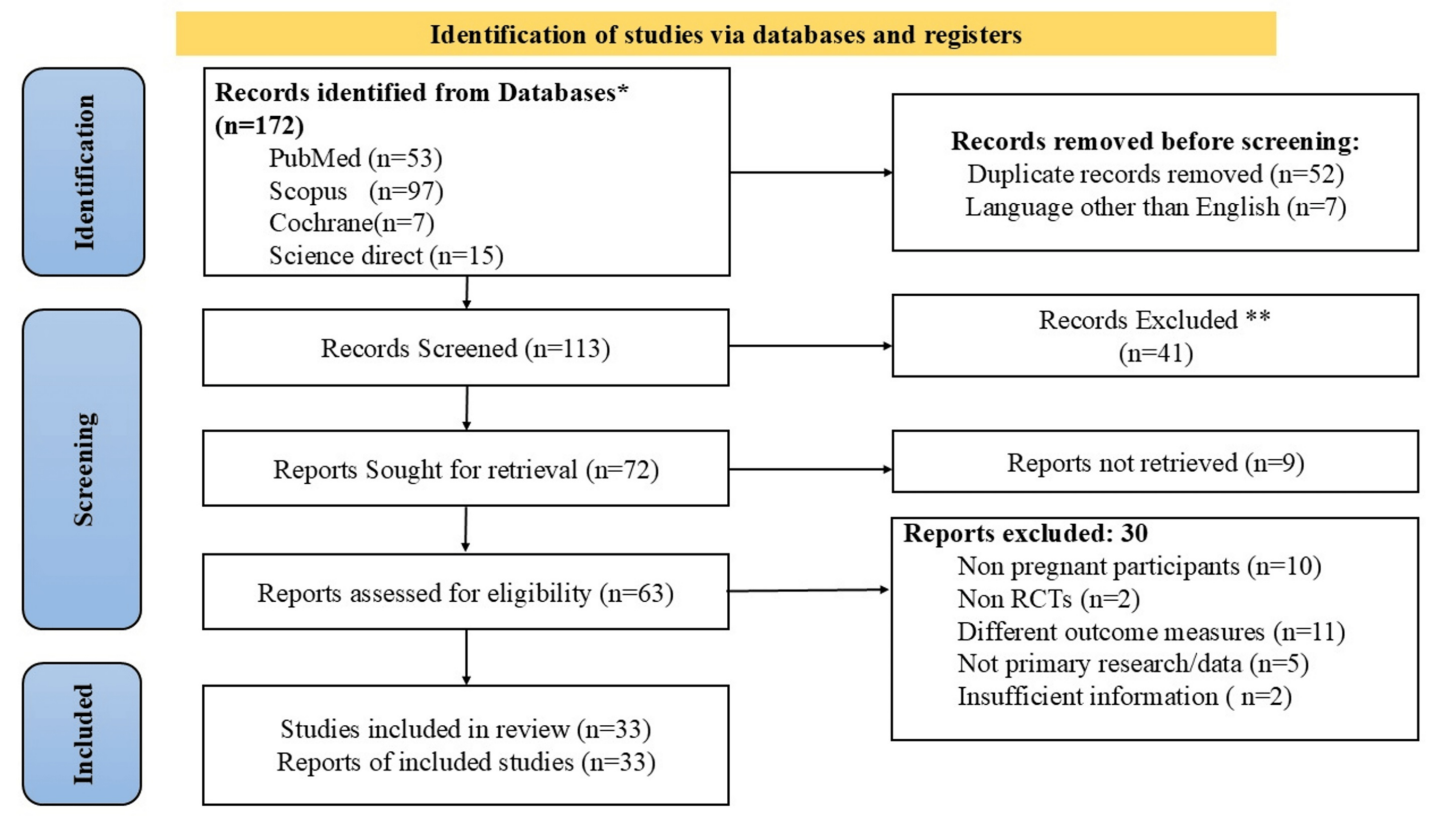
PRISMA 2020 flow diagram PRISMA: Preferred Reporting Items for Systematic reviews and Meta-Analyses

The systematic search and selection process yielded the following results: Initially, a total of 172 records were identified through electronic databases, manual searches, and other sources. After eliminating duplicates, 113 records underwent title and abstract screening, leading to the exclusion of records that did not meet the inclusion criteria. The remaining 64 records underwent a full-text assessment to ascertain their eligibility for inclusion. A careful evaluation against predefined inclusion and exclusion criteria resulted in 33 studies meeting the criteria for inclusion in the systematic review. The study selection process followed the Preferred Reporting Items for Systematic Reviews and Meta-Analyses (PRISMA) flow diagram as visually depicted in Figure 1. This diagram offers a clear overview of the number of records at each stage, including the reasons for exclusions.

Included Studies

Thirty studies appeared to meet the eligibility criteria but were excluded after screening the full text due to various reasons. Ten studies were excluded because they involved non-pregnant participants, participants trying to conceive, or participants undergoing procedures unrelated to pregnancy. Two of the studies were not randomized controlled trials, including qualitative studies, systematic reviews, and review articles. Eleven studies were excluded because they measured different outcomes, such as pain during unrelated medical procedures, psychosocial stress, or mental health outcomes. Additionally, few studies (two) lacked sufficient information to describe outcome measures or involved insufficiently described interventions. Five studies were excluded due to being letters to the editor or reviews of birth records rather than original research. The characteristics of the thirty-three studies that met the inclusion criteria and were ultimately included in the final analysis are detailed in Table 4. Out of the 33 RCTs, nine studies were from Turkey [15,18,26,27,39-41,43,46]. Four from Taiwan and the United States of America [19,20,32,33,21-23,45]. Three each from Iran [34,37,38]. Two each from Italy, Spain, and China [16,36,24,25,17,31]. And one each from Saudi Arabia, Thailand, Germany, Singapore, London, Finland, and Portugal [14,28,29,30,35,42,44]. This highlights the diversity of research contributions from various countries.

**Table 4:** Characteristics of included studies CS: cesarean; VAS: Visual Analog Scale; HAM-A: Hamilton Anxiety Scale; HAM-D: Hamilton Depression Scale; SBP: systolic blood pressure; DBP: diastolic blood pressure; STAI-S: State Anxiety Inventory; STAI-T: Trait Anxiety Inventory; SCDS: Satisfaction of Cesarean Delivery Scale; PSS; Perceived Stress Scale; EPDS: Edinburgh Postnatal Depression Scale; MSSCS: Maternal Satisfaction Scale for Cesarean Section' HRV: heart rate variability; BP: blood pressure; NST: Non-Stress Test; PIH: pregnancy induced hypertension; RR: respiratory rate; FHR: fetal heart rate; SAS: Self-Rating Anxiety Scale

Study Author (year)	Country	Inclusion Criteria	Sample Size/Sample	Intervention	Tool Used	Outcome Measures
Almedhesh et al., 2022 [14]	Saudi Arabia	Low-risk pregnant women undergoing elective CS with regional anesthesia. Parturient with normal vision and hearing abilities, no history of generalized anxiety disorder or mental illness, free from serious obstetrics complications (according to the obstetrician evaluation), and no increased intraoperative risk (such as placental disturbance) that was identified in the preoperative period.	351 (176 study and 175 control)	Virtual reality (Holy Quran verses with landscapes, music with landscapes)	Novel Visual Facial Anxiety Scale, Maternal Satisfaction Scale	Anxiety and stress among women undergoing CS under regional anesthesia.
Baltaci et al., 2022 [15]	Turkey	Hospitalization due to high-risk pregnancy, 19 years and older, pregnancy duration longer than 28 weeks, singleton pregnancy, duration of hospitalization 24 h minimum, ability to read and write and to comprehend, speaking Turkish.	76 (38 study group, 38 control group)	Lullaby recital (10 different lullabies, 2 min each)	Pregnant Information Form, the State Anxiety Inventory, and the Prenatal Attachment Inventory	Anxiety and prenatal attachment.
Buglione et al., 2020 [16]	Italy	Nulliparous singleton pregnancies, spontaneous labor, vertex presentation, 37-42 weeks gestational age.	30 women	Music therapy	Visual Analog Scale (VAS) for pain	Pain and anxiety during labor and delivery (active phase, second stage, 1h post-partum).
Cao et al., 2016 [17]	China	Patients with blood pressure over 130/90 mmHg or blood pressure elevation amplitudes of over 30/15 mmHg after 20 weeks of pregnancy.	60 patients with PIH	Music therapy	Hamilton Anxiety Scale (HAM-A) scores, Hamilton Depression Scale (HAM-D)	Pregnancy-induced hypertension treatment efficacy (SBP, DBP, HAM-A scores, HAM-D scores, serum angiotensin II levels).
Catalgol et al., 2022 [18]	Turkey	Pregnant women able to speak Turkish, ages 18-35, with no hearing impairment, and no smoking or drinking habits, applied for NST for the first time at 36 weeks of gestation, primipara, single, live and healthy fetuses.	100 pregnant women	12 instrumental classical Turkish Maqam Music listening at home and during NST application	State Anxiety Inventory (STAI-S), and Trait Anxiety Inventory (STAI-T)	Maternal anxiety and nonstress test findings.
Chang et al., 2005 [19]	Taiwan	Pregnant women between 20 and 40 years old, married, term pregnancies and planned to undergo cesarean births, if underwent spinal or epidural anesthesia, and normal newborns were singletons, with an Apgar score 7 at 5 minutes.	64 Women	Music therapy	Visual Analog Scale for Anxiety (VASA), Satisfaction of Cesarean Delivery Scale (SCDS)	Anxiety
Chang et al., 2008 [20]	Taiwan	Pregnant women aged 18 years or above expected to have uncomplicated vaginal deliveries with gestational age of 18–22 weeks (second trimester) or 30–34 weeks (third trimester).	236 (116 experimental group and 120 control group)	Four types of prerecorded music compact discs (CD) with 30 minutes of music consisting of lullabies	Perceived Stress Scale (PSS), State Scale of the State-Trait Anxiety Inventory (S-STAI) and Edinburgh Postnatal Depression Scale (EPDS)	Psychological health is measured by self-reported measures (PSS, S-STAI, EPDS).
Drzymalski et al., 2023 [21]	United States	Nulliparous women 18-50 years old with a healthy singleton pregnancy at ≥ 37 weeks gestational age undergoing elective cesarean delivery under neuraxial anesthesia.	20 parturient women	Mozart sonatas were broadcast to the music group immediately before patient entry and maintained throughout the procedure	Numeric Rating Scale, Maternal Satisfaction Scale for Cesarean Section (MSSCS)	Patient satisfaction changes in anxiety pre-and postoperatively. Postoperative means arterial pressure (MAP).
Drzymalski et al., 2017 [22]	Boston	Labouring participants and those scheduled for induction of labor aged 18 to 50 were eligible for enrolment;	100 parturient	Patients’ preferred music on a Pandora® station broadcast through an external amplified Speaker	Numeric Rating Scale (NRS)	The primary outcomes were 3 measures of anxiety. Secondary outcomes included pain, patient satisfaction, hemodynamic parameters, obstetric parameters, neonatal outcomes, and anesthesia provider anxiety.
Drzymalski et al., 2020 [23]	USA	Parturient women undergoing elective cesarean delivery.	150 parturient	Patient-selected or preselected music	Maternal Satisfaction Scale for Cesarean Section (MSSCS)	Preoperative anxiety during cesarean delivery: Postoperative anxiety, postoperative pain, and patient satisfaction.
Estrella-Juarez et al., 2023 [24]	Spain	Full-term pregnancy (≥37 weeks’ gestation) and low-risk pregnancy, nulliparity with a singleton pregnancy with no known fetal abnormality, no use of assisted reproductive technology, no perinatal complications, and no exposure to drugs or medications.	273 participants	Music therapy and virtual reality during NST and first stage of labor, 20 minutes each time	Spielberger State-Trait Anxiety Inventory, maternal blood pressure, maternal and fetal heart rates, and labor and birth outcomes	Anxiety, maternal and fetal physiologic parameters, labor and birth outcomes.
Garcia-Gonzalez et al., 2018 [25]	Spain	Nulliparous women in the third trimester.	409	Music therapy	Spielberger State-Trait Anxiety Inventory	Anxiety levels during pregnancy and fetal parameters.
Gokyildiz Surucu et al., 2018 [26]	Turkey	Primiparous women with term pregnancy (37-41 pregnancy weeks) without risk.	50 (25 each in experimental, and control group).	Music in Acemasiran mode for 3 hours during active labor	Pregnant Introductory Form, Evaluation Form on Labor, Visual Comparison Scale, STAI I-II State-Trait Anxiety Scale, Faces Anxiety Scale, Post-Delivery Evaluation Form	Pain perception anxiety level.
Handan et al., 2018 [27]	Turkey	Pregnant women with no communication and hearing problems, having cesarean indications, with less than 5 years between the first and the current cesarean section, no obstetric problems, and no known psychiatric diagnoses.	60 pregnant women	Songs chosen earlier by the patients were played during the cesarean section procedure	Questionnaire form, Visual Analog Scale (VAS)	Anxiety body temp, saturation, VAS, RR, HR, SBP, DBP.
Hanprasertpong et al., 2016 [28]	Thailand	Pregnant women between 15 and 21 weeks of gestation who underwent a second-trimester genetic amniocentesis due to advanced maternal age.	332 (161 experimental, 171 controls)	Music listening	Visual Analog Scale (VAS)	Pre-procedure anxiety, the anticipated pain, post-procedure pain/ anxiety median VAS scores, pain rating, future decision and level of pain compared to a venipuncture.
Hepp et al., 2018 [29]	Germany	Pregnant women with an indication for primary cesarean, regional anesthesia, having German language comprehension, normal hearing, no serious comorbidities.	304 patients	Music had four self-selected genres. At admission, at skin incision, during skin suture, and two hours after completion of surgery	State-Trait Anxiety Inventory, Visual Analog Scale for Anxiety	Stress, anxiety and objective parameters (salivary cortisol/amylase, heart rate, blood pressure).
Kakde et al., 2023 [30]	Singapore	Parturient aged 21 to 50 years old (ASA) physical status 2, undergoing elective cesarean delivery under spinal anesthesia.	108 parturient	Music listening	Visual Analog Scale – Anxiety (VAS-A)	Perioperative anxiety, acute pain and pain catastrophizing.
Li et al., 2012 [31]	China	Women undergoing cesarean delivery aged from 20 to 35 years with no comorbidities.	60 (study group-30, control group-30)	Slow-rhythm music for 30 minutes before surgery of heart rate variability	Self-Rating Anxiety Scale and a Visual Analog Pain Scale	Anxiety and differences in SAS scores and HRV values.
Liu et al., 2010 [32]	Taiwan	Normal term pregnancy with singleton fetus gone planned for a vaginal delivery without using pharmacological analgesia during labor.	60 primiparas	Music therapy with self-selected music during labor	Self-report Visual Analog Scale	Labour pain and anxiety.
Liu, et al., 2016 [33]	Taiwan	Pregnant women above 30 years of age with 18-34 weeks of gestation, with disturbed sleep.	121 women	Music listening	Pittsburgh Sleep Quality Index (PSQI) score, Patient Satisfaction Scale (PSS), STAI	Stress, anxiety, and sleep quality for sleep-disturbed pregnant women.
Momeni et al., 2020 [34]	Iran	Mothers aged 18 to 35 years with a gestational age of 37 to 42 weeks, fetal weights of 2500 to 3500 g, and absence of known medical conditions, and allergies.	130 women	Snoezelen's room was designed using an aquarium, and a projector, which played optical shapes, light music, and essential aroma	Demographic characteristics form, Harman’s Childbirth Attitude Questionnaire (CAQ), Visual Analogue Scale (VAS), and the Mackey Childbirth Satisfaction Rating Scale	Anxiety and maternal satisfaction.
Nwebube et al., 2017 [35]	London	Pregnant women over 18 years From English-speaking countries were eligible.	111 participants	Music group (daily listening to specially composed songs) or a control group (daily relaxation) for 12 weeks each.	State and Trait Anxiety (Spielberger), (Edinburgh Postnatal Depression Scale (EPDS)	Prenatal anxiety and depression.
Parodi et al., 2021 [36]	Italy	Women with a low-risk pregnancy with 37 weeks" gestation undergoing elective cesarean section.	60 women	Novel binaural beat technique	State-Trait Anxiety Inventory (STAI-Y) questionnaire	Preoperative anxiety.
Reza et al., 2007 [37]	Iran	Pregnant women (ASA I) scheduled for elective cesarean section under general anesthesia.	100.	Intraoperative music by CD-player	Visual Analog Scale (VAS)	Postoperative pain morphine requirement, anxiety, and vomiting.
Rezaei et al., 2023 [38]	Iran	Pregnant women aged 18-35 years, 32-41 weeks with wanted pregnancy, stable hemodynamic status, and no meals at least 2 hours prior.	195	Lavender oil inhalation and music therapy	State-Trait Anxiety Inventory (STAI)	Anxiety and NST results.
Simavli et al., 2014 [39]	Turkey	Primiparous women aged 18 to 35 years, 37–41 weeks of gestation with a singleton pregnancy, cephalic presentation, and normal birth weight, expected to have normal spontaneous delivery.	161	Self-selected music during labor	Visual analog scale (VAS), EPDS	Postpartum pain and anxiety satisfaction and postpartum depression.
Simavli et al., 2014 [40]	Turkey	Primiparous women aged between 18 and 36 years at 37–41 weeks of gestation with singleton pregnancies and babies of cephalic presentation, expected normal spontaneous delivery and maternal.	156 primiparous women	Music therapy	Visual Analog Scale (VAS), Edinburgh Postpartum Depression Scale	Labor pain and anxiety, maternal-fetal parameters and analgesic requirement.
Soylu et al., 2022 [41]	Turkey	Pregnant women aged 18-45 years, with 32 or more weeks of gestation having no fetal anomalies and no communication obstacle.	74 pregnant women	Music therapy	Pregnant Information Form, NST Result Form, and the State and Trait Anxiety Inventory	Fetal wellbeing through NST, anxiety levels and vital signs of pregnant women.
Teckenberg-Jansson et al., 2019 [42]	Finland	Pregnant women aged 18–47 years, hospitalized due to pregnancy-related complications.	102 pregnant women	Live music therapy	PSS, STAI	Heart rate variability and self-reported stress and anxiety.
Toker et al., 2017 [43]	Turkey	Pregnant clients with a hospital stay of five days and diagnosis of preeclampsia, within 30th or more weeks of pregnancy.	70 pregnant women	Turkish classical music therapy trials	The Personal Information Form, State-Trait Anxiety Inventory, and Newcastle Satisfaction with Nursing Scale	Anxiety scores satisfaction fetal movement counts, FHR & BP.
Ventura et al., 2012 [44]	Portugal	Pregnant women with gestational age between 110 and 165 days having singleton spontaneous pregnancies.	154 pregnant women	Relaxing music intervention for 30 minutes while sitting and reading magazines	Spielberger’s State and Trait Anxiety Inventory	Plasma cortisol self-reported state anxiety score.
Wu et al., 2012 [45]	United States	Participants aged 18 to 50 years verbally fluent in English and undergoing an elective surgical abortion.	26 women	Music listening	State-Trait Anxiety Inventory (STAI), Verbal Numerical Scales	Pain, anxiety and coping patient satisfaction.
Yüksekol et al., 2020 [46]	Turkey	Pregnant literate women with a single and living fetus; gestational age of 28–32 weeks; hospitalized due to mild preeclampsia.	60	Music therapy	Introductory and Evaluation Form, the Blood Pressure Form, and the State Anxiety Inventory	Arterial blood pressure and anxiety levels.

The key findings of all the included studies are presented in (Table 5)

**Table 5:** Key findings NST: Non-Stress Test; STAI-S: State Anxiety Inventory; EPDS: Edinburgh Postnatal Depression Scale

Reference	Intervention	Key Findings
Almedhesh et al. 2022 [14]	Participants used VR glasses during and after regional anesthesia, choosing between Quran recitations with natural landscapes or relaxing music.	VR group had significantly lower stress and anxiety levels immediately after surgery and 2 hours postoperative (p=0.000). Significant differences were found in anxiety levels compared to the control group at various points during the procedure.
Baltaci et al., 2022 [15]	The intervention group listened to 10 lullabies for 20 minutes while touching their abdomen, compared to standard care in the control group.	Post-intervention, the intervention group had significantly lower anxiety levels and higher prenatal attachment compared to the control group
Buglione et al., 2020 [16]	The intervention group listened to music (three preferred tune categories: gentle popular music, soft classical music, and Israeli tunes) during labor compared to standard care in the control group. Participants were given the freedom to choose songs based on their preferences.	The intervention group reported lower pain levels during the active phase of labor and 1 hour postpartum, and lower anxiety levels during labor and postpartum. Notably, there was no statistically significant difference between the groups in terms of pain level during the second stage of labor.
Cao et al., 2016 [17]	The observation group received music therapy for 30 to 60 minutes each day over four weeks alongside conventional treatment. The musical choices predominantly included folk music and symphonies by renowned composers such as Beethoven, Schubert, and Tchaikovsky, along with the patient's preferred slow and deliberate songs. The therapy sessions commenced two hours after breakfast and dinner.	The observation group had lower anxiety and depression scores, higher quality of life scores, and lower serum Ang II levels post-treatment compared to the control group.
Catalgol et al., 2022 [18]	Pregnant women listened to Classical Turkish Maqam Music during NST and at home as per their choice in the 36th, 37th, and 38th weeks of pregnancy. The women had the flexibility to adjust the volume according to their preferences and also had the option to choose their preferred music from the set of 12 songs.	The intervention group had significant reductions in anxiety levels during NST applications at various gestational weeks compared to pre-application levels and the control group. No difference between the groups was observed in terms of anxiety scores assessed on the first postpartum day (p > 0.05). Additionally, the mean score of the intervention group was higher than the control group at 36 and 38 weeks of gestation (p < 0.001).
Chang et al., 2005 [19]	Participants chose calming music genres including Western classical, new age, or Chinese religious music to listen to during the cesarean section via earphones, a portable compact disc player, and a personalized music disk for the cesarean section. They listened to music for a minimum of 30 minutes, starting from the initiation of anesthesia until the conclusion of surgery. Participants in the control group received standard nursing care.	The experimental group exhibited significantly lower anxiety levels and a higher satisfaction level concerning the cesarean experience. There were no noteworthy differences between the two groups in any of the physiological indicators.
Chang et al., 2008 [20]	Prerecorded music CDs featuring lullabies, classical music, nature sounds, and Chinese children's songs were used at a tempo to emulate the human heart rate (60–80 beats/minute).	The music therapy group had a notable reduction in stress and anxiety scores post-intervention compared to the control group. On average, women in the experimental group tended to have lower scores than the control group, with a difference of 2.63 units.
Drzymalski et al., 2023 [21]	Music (Mozart sonatas) was played during the surgical procedure through an iPod using an amplified speaker, just before the patient's entry and persisted throughout. In contrast, the control group did not partake in music listening, and any existing radio in the operating room was switched off before the patient entered.	No significant differences in overall patient satisfaction, anxiety change, or postoperative mean arterial pressure between music and control groups.
Drzymalski et al., 2017 [22]	Patients listened to preferred music during labour analgesia, compared to no music in the control group. The music was played at a standardized medium volume, adjusted according to patient preferences, and utilized the commercial-free version (Pandora® One) with explicit content disabled. To ensure a standardized sound environment, the labor room door was closed, the television was turned off, and extraneous sounds were minimized.	The music group had higher anxiety immediately after epidural catheter placement and lower relaxation levels compared to the control group.
Drzymalski et al., 2020 [23]	Participants listened to chosen music from Pandora or Mozart sonatas during and after surgery whereas the control group did not have any music exposure. The music initiation occurred in the preoperative area, continued throughout the entire procedure in the operating room (OR), including spinal placement, and persisted for one hour after reaching the postoperative area. The total duration of music exposure in the Pandora and Mozart groups was 186 ± 50 minutes and 193 ± 49 minutes, respectively.	Anxiety levels were lower in the Mozart group compared to the control group, but no significant differences were found in postoperative anxiety levels between the Pandora and control groups.
Estrella-Juarez et al., 2023 [24]	In the music intervention group, participants were provided with an iPod equipped with wireless headphones for isolated listening experiences during the Non-Stress Test (NST) and the first stage of labor, each lasting 20 minutes. Music Group: "Musical Journey through Pregnancy" with iPod and headphones; Virtual Reality (VR) intervention group: 3D ocean images and soothing sounds.	Significant reductions were seen in total and trait anxiety for both music and VR groups. Conversely, the control group displayed minimal alterations in total anxiety, trait anxiety, and state anxiety levels with no statistically significant differences.
Garcia-Gonzalez et al., 2018 [25]	Pregnant participants underwent a 40-minute music therapy intervention, engaging with the CD "Musical Journey through Pregnancy" by Gabriel F. Federico in sessions held three times a week at a consistent time for a total of 14 sessions. The intervention, which excluded sleep, continued until the 36th week, when participants were scheduled for a Non-Stress Test (NST) at the Obstetrics hospital unit, incorporating the ongoing music therapy intervention.	Lower state and trait anxiety post-NST in the music group compared to the control group. In contrast, the control group's STAI-S scores remained consistent before and after the NST
Gokyildiz Surucu et al., 2018 [26]	Expectant mothers engaged in music listening in the Acemasiran mode for 3 hours (with 20 minutes of listening followed by 10 minutes of resting) using earphones, initiated when their dilation reached 4 cm and progressed into the active phase of labour. The control group underwent no additional interventions beyond routine procedures.	Post-intervention, there was a significant reduction in both state anxiety scores and Faces Anxiety Scale scores (p<0.05) for music group women who reported lower levels of pain, reduced anxiety, perceived the labor as easier, experienced longer contraction periods, and observed a faster progression in their labor.
Handan et al., 2018 [27]	Preoperatively selected songs were played during the entire operation at each patient's preferred volume using a stereo player.	The experimental group exhibited significantly lower VAS scores before and during the procedure, reduced body temperature, anxiety, and blood pressure; and increased oxygen saturation both pre- and postoperatively.
Hanprasertpong et al., 2016 [28]	The intervention involved exposing participants undergoing amniocentesis to Thai classical music composed by King Bhumipol, using earphones throughout antiseptic skin preparation to the needle removal stage.	Listening to music did not show a statistically significant impact on reducing post-procedure pain or anxiety. No statistically significant differences in subjective pain ratings, the inclination to undergo a similar procedure, or the perceived pain level compared to venipuncture.
Hepp et al., 2018 [29]	The music intervention for the music group commenced upon the participant's entry into the operating theatre continuously played at a standardized volume of 55 dB (A) measured at the participants' head. The control group, in contrast, did not receive any music at admission, during skin incision, during skin suture, or two hours after completing the surgery.	The music group exhibited significantly lower anxiety levels, and increased satisfaction, than the control group at the skin suture point However, there was no significant difference 2 hours after surgery .89.7% found the music made the situation more enjoyable, and 73.4% believed the music had a calming effect.
Kakde et al., 2023 [30]	Parturient in the experimental group participated in music listening for approximately 30 minutes, via portable Bluetooth speaker starting during the administration of spinal anesthesia and continuing through the cesarean delivery. Following the surgery, the parturient continued their music-listening experience for another 30 minutes in the post-anesthesia care unit (PACU) by earphones.	Music listening correlated with notably reduced postoperative anxiety and improved patient comfort and satisfaction scores. feedback; However no substantial difference in acute pain scores was observed.
Li et al., 2012 [31]	In the intervention group, participants chose Chinese classical music, closed their eyes, and relaxed for 30 minutes in a quiet environment before surgery, listening to the selected music.	After the procedure, the study group exhibited significantly lower anxiety, reduced postoperative pain scores, and improved heart rate variability while the control group's score remained unchanged.
Liu et al., 2010 [32]	Participants listened to calming, anxiety-reducing music for a minimum of 30 minutes during both the latent phase (2–4 cm cervical dilation) and active phase (5–7 cm cervical dilation) of labor.	Women in the music-listening group experienced lower pain and anxiety levels and higher finger temperatures compared to their counterparts in the control group. However, no significant interactions were observed on all outcome measures during the active phase.
Liu, et al., 2016 [33]	Participants had daily music listening, at least a 30-minute session from their preferred music or precompiled compact discs (CDs) crafted by the researcher at bedtime over two weeks. Notably, the musical tempo aligned with the human heart rate, ranging from 60 to 80 beats per minute.	The experimental group demonstrated significantly lower reduced anxiety, and stress and improved sleep quality compared to the control group.
Momeni et al., 2020 [34]	The intervention involved creating a calming environment (Snoezelen's room has an aquarium, a projector displaying soothing optical shapes, ambient light music, and the use of essential aroma).	In the active phase, the intervention group exhibited decreased anxiety especially at 6 – 7cm and 7 - 8 cm of dilation scores compared to the control group. Furthermore, post-delivery, reduced anxiety was noted in the intervention group while in the control group, increased anxiety was observed.
Nwebube et al., 2017 [35]	The music group participants engaged in daily listening to specially composed songs whereas the control group engaged in daily relaxation for minimum 20 minutes for 12 weeks.	The music group exhibited significantly lower Trait Anxiety, State Anxiety, and EPDS (depression) scores at week 12 compared to baseline.
Parodi et al., 2021 [36]	The intervention involved utilizing a binaural-based technique (BB) i.e. auditory illusion by presenting two distinct pure-tone sine waves dichotically, with each entering one ear using the Dynamic Multi Spectrum Phase Shift (DMSPS) algorithm for deep relaxation. The algorithm dynamically modulates band shifting to generate BB, specifically targeting brainwave patterns associated with deep states of relaxation observed during activities like yoga and meditation sessions.	Significant reduction in state anxiety with binaural music compared to control and normal music groups.
Reza et al., 2007 [37]	Patients in the intervention group received intraoperative music (Spanish-style guitar)via CD. Conversely, the control group was exposed to a blank CD. To ensure an immersive experience, all patients wore headphones that fully covered their ears, preventing any external sounds from the operating room.	No statistically significant difference was observed between the two groups in terms of postoperative anxiety scores, vomiting frequency, morphine requirements, and pain scores up to six hours after the surgery (P>0.05).
Rezaei et al., 2023 [38]	The lavender/aromatherapy group experienced the inhalation of four drops of 10% lavender oil applied to a polyethylene napkin, secured to their clothing. The lavender/aromatherapy-music group additionally also listened to nature sounds, such as waterfalls and birds, through wireless earphones for 20 minutes. The control group received a placebo, inhaling distilled water under the same conditions before NST.	Significant reduction in state anxiety for both lavender/aromatherapy and lavender/aromatherapy-music groups in comparison to the control group.
Simavli et al., 2014 [39]	Participants chose from six types of music with relaxing patterns including classical music, light music, popular music, Turkish art music, Turkish folk music, and Turkish Sufi music. Women were allowed to choose whether or not to use headphones. The tempo of the music was selected to mimic the human heart rate (60–80 beats/min).	Anxiety levels were significantly lower in the music therapy group compared to the control group at all time intervals (p < 0.001), with higher satisfaction rates. Reduced postpartum anxiety and pain, increased satisfaction during labor, and diminished postpartum depression rates were noted in the e-music therapy group.
Simavli et al., 2014 [40]	Five music genres—classical music, Turkish art music, Turkish folk music, Turkish classical music, and popular music—were delivered through headphones based on the participant's choice, encompassing the most popular music types in the region. Meanwhile, the control group participants were exposed to a blank CD during labor. The intervention commenced once participants reached 2 cm cervical dilatation.	Lower mean anxiety and pain scores in the music group throughout the first (all phases) and second stage of labor, and postpartum compared to the control group (p < 0.001). Moreover, a noteworthy finding was the significant difference in maternal hemodynamic and fetal heart rates between the two groups after the intervention (p < 0.01).
Soylu et al., 2022 [41]	Pregnant women listened to their preferred music via earphones and an MP3 player during the NST procedure lasting approximately 30 minutes.	Post-procedure anxiety scores were significantly lower in the music group compared to the control group (p < 0.001).
Teckenberg-Jansson et al., 2019 [42]	The music therapy group received live sessions over three days, featuring lyre playing and humming by a trained therapist. The pentatonic lyre was played on the abdomen, accompanied by humming lullabies. Participants were encouraged to join in, and some had the opportunity to play the lyre.	Significant reduction in state anxiety in both music therapy and control groups; no significant difference between groups.
Toker et al., 2017 [43]	Participants engaged in a daily 30-minute music-listening session for seven days through an MP3 player and headphones while lying down.	No statistically significant differences in anxiety levels were observed between the groups before and after five days (p > 0.05). However, higher satisfaction with nursing care noted in the music group
Ventura et al., 2012 [44]	Four types of music compact discs (CDs) with approximately 30 minutes of music. A 3-minute demonstration CD, featuring excerpts from the four types of music, was also prepared and played for the participants in the music group, allowing them to choose the type they found most relaxing.	A significant anxiety reduction was observed for the music group compared to reading a magazine or waiting in a waiting room.
Wu et al., 2012 [45]	Participants chose from five preloaded music tracks using a portable media player (iPod®; Apple) with earphones equipped with disposable sanitary covers.	The music group reported a lower anxiety score (after speculum removal) in comparison to the control group. After adjusting for baseline factors such as anxiety, depression, gestational age, parity, or previous abortions, no significant group differences in pain or anxiety scores were identified.
Yüksekol et al., 2020 [46]	Participants in the intervention group, listened to music for 30 minutes in the morning and evening, adopting a comfortable position in their beds within their respective rooms. In contrast, the control group did not engage in any music listening.	Lower anxiety was reported in the intervention group after both morning and evening music listening sessions compared to the control group (p < 0.05).

Study Participants/Study Size and Design

This review includes data from 4363 pregnant women who participated in the 33 included RCTs. The included studies generally focus on pregnant women who meet specific criteria, varying by country and study design. Most studies included women within a certain age range, typically between 18 and 50 years old, and often specified gestational age, usually between 28 and 42 weeks. Many studies required participants to be undergoing a specific type of delivery, such as elective cesarean section or normal vaginal delivery, often under particular anesthesia conditions. Other common criteria included singleton pregnancies, the absence of serious obstetric complications, and the ability to communicate in the language of the study country.

In several studies, additional inclusion criteria were related to health conditions or risks, such as high blood pressure, preeclampsia, or hospitalization due to pregnancy-related complications. Some studies also specified that participants should have no known medical conditions, comorbidities, or psychiatric diagnoses, ensuring a relatively homogeneous sample of low-risk pregnancies. A few studies focused on women with specific needs or undergoing particular procedures, such as amniocentesis or surgical abortion. Across all studies, there was a clear emphasis on ensuring that participants were able to understand and consent to the research, often requiring literacy and verbal fluency in the study language.

Intervention

The studies included in this review used a variety of music interventions, from listening to prerecorded music to live music therapy. Some studies focused on specific types of music, such as lullabies, classical Turkish Maqam music, Mozart sonatas, and genres chosen by the patients themselves. Music therapy was sometimes combined with other elements like virtual reality, the recitation of the Holy Quran, or calming visual scenes. In some cases, patient-selected or preselected music was played during medical procedures, such as cesarean sections or labor. Other studies used slow-rhythm music, relaxing music, or innovative approaches like binaural beats.

In some interventions, music was paired with other sensory experiences, such as the inhalation of lavender oil or using a Snoezelen room with light music and visual effects. The duration of these music interventions varied, with some lasting only during specific medical procedures, while others involved daily listening sessions over several weeks. Music was sometimes played through headphones or speakers, and in other instances, it was part of a multi-sensory experience involving visual or tactile elements. The types of music ranged from classical pieces and lullabies to cultural and patient-selected genres, with intervention durations varying from 20 minutes during non-stress tests to several hours during labor. The goal of these interventions was to reduce anxiety, promote relaxation, and improve the overall patient experience, with careful attention to the environment and patient preferences.

Duration of Trials

Across the 33 studies reviewed, the duration of music intervention varied significantly depending on the context and objectives. The intervention durations ranged from brief sessions lasting 20 to 30 minutes to more extended periods of continuous music exposure during entire surgical procedures or labor. In some studies, participants listened to music for 20-40 minutes during specific procedures like the non-stress test (NST) or labor. In contrast, other interventions involved continuous music from the initiation of anesthesia until the conclusion of surgery, which could last several hours. Several studies implemented daily or repeated sessions of music therapy, lasting from 20 to 60 minutes per session, over days, weeks, or even throughout the pregnancy. For instance, some interventions involved daily music sessions for 30 minutes over one week or up to 12 weeks, with participants controlling the volume and timing. The interventions were designed to align with the natural rhythms of the heart, aiming to create a relaxing environment tailored to the participant's needs, whether in the context of surgery, labor, or prenatal care.

Tools/Instruments Used

The tools used in the studies were primarily focused on assessing anxiety and stress among participants employing scales like the Visual Analog Scale (VAS) for anxiety, the State-Trait Anxiety Inventory (STAI), and the Hamilton Anxiety and Depression Scales (HAM-A and HAM-D). Several studies also utilized the Edinburgh Postnatal Depression Scale (EPDS) and the Perceived Stress Scale (PSS). Maternal satisfaction was measured using various tools, such as the Maternal Satisfaction Scale for Cesarean Section (MSSCS) and the Satisfaction of Cesarean Delivery Scale (SCDS). Additionally, some studies incorporated self-report tools like the Pittsburgh Sleep Quality Index (PSQI) and specific anxiety scales like the Faces Anxiety Scale and the Visual Facial Anxiety Scale. These tools were often complemented by demographic questionnaires, evaluation forms, and other context-specific instruments to assess the overall experience and outcomes of the participants.

Outcomes Measured

The primary outcomes measured across the studies were anxiety and stress levels among pregnant women, particularly those undergoing cesarean sections or labor. These were assessed at various stages: before, during, and after procedures with a range of validated tools.

Secondary outcomes included pain levels during labor and postoperatively, patient satisfaction, and various physiological parameters such as heart rate, blood pressure, and salivary cortisol levels. Additional secondary outcomes focused on maternal and fetal well-being, including heart rate variability, fetal heart rate, and maternal blood pressure. Other studies also measured psychological health indicators such as prenatal attachment, depression, and sleep quality. Obstetric and neonatal outcomes, analgesic use, and postpartum parameters were also evaluated as part of the secondary outcomes in these studies.

The settings for music interventions in the study varied across different environments and approaches. Participants experienced music through various devices like VR glasses, MP3 players, CD players, iPods, and stereos in clinical settings, such as during surgeries, labor, or prenatal testing, and were tailored to specific patient needs.

Risk of Bias in Included Studies

The methodological quality assessment of included studies using the Joanna Briggs Institute (JBI) tool evaluated bias related to selection and allocation, administration of intervention/exposure, assessment, detection, measurement of the outcome, participant retention, and also the validity of statistical conclusion validity. The evaluation was done for thirteen parameters to the best of the investigator's knowledge and understanding and included the following questions: was true randomization used for the assignment of participants to treatment groups; was allocation to treatment groups concealed; were treatment groups similar at the baseline; were participants blind to treatment assignment; were those delivering the treatment blind to the treatment assignment; were treatment groups treated identically other than the intervention of interest; were outcome assessors blind to treatment assignment; were outcomes measured in the same way for treatment groups; were outcomes measured in a reliable way; was follow-up complete, and if not, were differences between groups in terms of their follow-up adequately described and analyzed; were participants analyzed in the groups to which they were randomized; was appropriate statistical analysis used; was the trial design appropriate, and were any deviations from the standard RCT design (individual randomization, parallel groups) accounted for in the conduct and analysis of the trial?

Most of the thirty-three studies demonstrated high-quality standards, with the majority scoring "yes" on most of the items. This suggests that the studies generally adhered to recommended research protocols, providing a strong basis for reliable findings. Studies such as Catalgol et al., 2022, Estrella-Juarez et al., 2023, Hanprasertpong et al., 2016, Parodi et al., 2021, Reza et al., 2007, Simavli et al., 2014 scored "yes" across all items, indicating robust methodology and reporting standards [18,24,28,36,37,39,40]. Nonetheless, some studies had unclear or negative responses on several items, indicating potential limitations in their design or reporting. For example, Baltaci et al., 2022, and Drzymalski et al., 2023, had "no" or "unclear" on items like Item 4 (allocation concealment) and Item 5 (blinding of participants), suggesting possible biases in their findings [15,21]. Item 5 (blinding of participants) and Item 6 (blinding of outcome assessors) were often marked as "unclear" or "no," indicating a frequent issue with blinding, which can impact the objectivity of the results. Studies with multiple unclear responses, such as Cao et al. (2016), Li et al. (2012), and Liu et al. (2010), indicate areas where the research design or reporting might lack transparency or rigor [17,31,32].

Variations across studies: Variability in scoring across studies highlights the diversity in research design and reporting quality. For instance, while some studies had robust methodologies, others showed significant weaknesses, especially in areas like blinding and allocation concealment. The variation in the quality of the studies suggests that while some findings can be considered reliable, others should be interpreted with caution. Future research could benefit from improved blinding procedures and more transparent reporting to enhance the overall quality and reliability of evidence in the field. The overall data reflects a mix of high-quality and moderate-quality studies, with common areas of concern related to blinding and allocation concealment. The consistently high scores in other areas suggest a generally strong evidence base, though care should be taken in interpreting findings from studies with multiple "no" or "unclear" responses.

Effect of interventions

In reviewing the effectiveness of various interventions for reducing anxiety and improving patient outcomes, several approaches have proven effective. Virtual reality (VR) was notably effective, as highlighted by Almedhesh et al. (2022), which showed significant reductions in stress and anxiety levels [14]. Music therapy emerged as another highly effective intervention, with studies such as Cao et al. (2016), Catalgol et al. (2022), Chang et al. (2008), Gokyildiz Surucu et al. (2018), Hepp et al. (2018), Kakde et al. (2023), Li et al. (2012), Liu et al. (2010), Liu et al. (2016), Momeni et al. (2020), Nwebube et al. (2017), Reza et al. (2007), Simavli et al. (2014), Simavli et al. (2014), Soylu et al. (2022), Ventura et al. (2012), Wu et al. (2012), Yüksekol et al. (2020) demonstrating substantial reductions in anxiety and pain, along with increased patient satisfaction [17,18,20,26,29-35,37,39-41,44-46]. Aromatherapy, specifically using lavender, was also beneficial, as indicated by Rezaei et al. (2023) [38]. Binaural music showed effectiveness in reducing state anxiety, according to Parodi et al. (2021) [36]. Music intervention also helped in improving prenatal attachment (Baltaci et al., 2022) [15].

Conversely, some interventions did not achieve significant effects. For example, Drzymalski et al. (2023), Drzymalski et al. (2020), Hanprasertpong et al. (2016), and Toker et al. (2017) found no notable benefits of music in reducing pain or anxiety [21,23,28,43]. Additionally, certain music therapy studies, such as those by Drzymalski et al. (2017) and Teckenberg-Jansson et al. (2019), did not show significant differences in outcomes compared to control groups [22,42]. Overall, while many interventions proved effective, the variability in results highlights the need for further research to optimize these approaches.

Quality of Evidence

The quality of evidence for the included studies was assessed using the Grading of Recommendations, Assessment, Development, and Evaluation (GRADE) system. In this review, the quality of evidence varied across the studies, with some demonstrating high-quality evidence due to robust study designs, large sample sizes, and consistent results. Catalgol et al., 2022; Estrella-Juarez et al., 2023; Hanprasertpong et al., 2016; Parodi et al., 2021; Reza et al., 2007; Simavli et al., 2014; Simavli et al., 2014 [18,24,28,36,37,39,40] However, several studies were rated as having low or very low-quality evidence: Cao et al., 2016, Li et al., 2012, and Liu et al., 2010 [17,31,32]. This was often due to limitations such as small sample sizes, risk of bias, or heterogeneity in study design and outcome measures.

Many of the included studies were downgraded due to issues such as imprecise estimates, potential publication bias, or inconsistency in the findings. For instance, while some studies provided strong evidence for the effectiveness of music interventions in reducing maternal anxiety and pain, others showed mixed or inconclusive results, leading to a lower overall quality rating and demanding further research to strengthen the conclusions. This variability in the quality of evidence underscores the need for more rigorous, large-scale studies to better understand the impact of music interventions on maternal well-being.

Discussion

The researchers identified 33 randomized controlled trials that used music as an intervention for pregnant women at different stages of pregnancy. These studies evaluated the effects of music on pain and anxiety levels. The review covers a range of methodologies, including music therapy, virtual reality (VR), and other relaxing interventions.

By combining the findings from these various studies, the discussion aims to add to the growing body of knowledge on non-pharmacological approaches to improving maternal well-being. The outcomes were assessed at different time points, and the findings are organized under the following headings:

Maternal Anxiety Reduction

Maternal anxiety and stress, especially during the perinatal period, can be significantly alleviated through music therapy. The calming effects of music can help lower cortisol levels and activate the parasympathetic nervous system, which promotes relaxation. This is particularly useful during labor, as reducing anxiety can help lessen the perception of pain and may even shorten the duration of labor [47,48]. Music therapy provides several benefits for women during the childbirth process. It helps them relax, lowers stress, improves emotional well-being, and offers comfort and a sense of connection. By creating a soothing auditory environment, music therapy can shift the mother's focus away from the stress and pain of childbirth, contributing to a more positive birthing experience [49]. Additionally, music can evoke emotional responses that allow mothers to express and process their fears and anxieties, further reducing overall stress [50].

A comprehensive review of studies highlights the effectiveness of music therapy in reducing maternal anxiety during various stages of labor. For example, Buglione (2020) reported that music therapy during labor led to lower pain levels in the active phase and significantly reduced anxiety levels during different stages of labor and postpartum [16]. Similarly, Gokyildiz Surucu (2018) found that women who listened to music in the Acemasiran mode during labor experienced reduced pain and anxiety, perceived labor as easier, and observed a faster progression [26]. Liu (2010) showed that women in the music-listening group had lower pain and anxiety levels, as well as higher finger temperatures, compared to the control group during the latent phase of labor [32]. Simavli (2014) demonstrated that music therapy significantly reduced anxiety levels throughout all stages of labor [39]. A finding consistent with Momeni (2020), who also observed a significant reduction in anxiety during the active phase of labor and lower anxiety scores post-delivery compared to the control group [34]. Estrella-Juarez’s (2023) study on music and virtual reality (VR) interventions during non-stress tests (NST) and labor showed significant reductions in total and trait anxiety levels in both intervention groups, with no significant changes in the control group [24].

Beyond labor, music therapy has shown the potential to reduce anxiety in both preoperative and postoperative settings for cesarean sections and other obstetric procedures. Research indicates that listening to music before surgery can significantly lower preoperative anxiety, which is essential for better surgical outcomes and faster recovery [51]. Additionally, music therapy after surgery can help reduce stress, promote relaxation, and enhance the overall emotional well-being of the mother [52]. The review reveals various studies reporting significant reductions in anxiety during cesareans, like Chang (2005) and Handan (2018), which noted significantly lowered anxiety levels in the experimental group compared to the control group [19,27]. Participants also reported higher satisfaction with their cesarean experience, although there were no significant physiological differences between the groups. Almedhesh's (2022) research compared the effects of virtual reality (VR) and music therapy and observed that the VR group experienced significantly lower stress and anxiety levels immediately after skin suturing and two hours postoperatively (p=0.000) supported by mixed factorial ANOVAs, indicating significant differences between the VR and control groups in terms of time, group, and time-group interaction (p=0.000 for all comparisons) [14]. Hepp's research (2018) showed significantly lower anxiety at the skin suture point and two hours post-surgery, with significant effects on both measurement time point and group [29]. Kakde (2023) disclosed significantly reduced postoperative anxiety scores and Perceived Control Scale (PCS) scores [30]. Participants also provided feedback that the music helped reduce anxiety and had a calming effect. Li (2012) reported significantly lower anxiety scores (SAS scores), which remained lower six hours post-surgery compared to those in the control group [31].

Multiple studies (e.g., Baltaci, 2022; Cao, 2016; Catalgol, 2022) revealed that music therapy groups consistently showed lower anxiety levels compared to control groups who did not receive any form of music intervention [15-17]. For example, in Baltaci's study, the intervention group displayed significantly lower anxiety levels after a two-day lullaby intervention, while the control group showed no significant changes. This consistent trend across studies supports the notion that music therapy can provide a significant reduction in anxiety levels compared to no intervention.

Some studies also explored the long-term effects of music therapy. Chang's studies from 2005 and 2008, for instance, demonstrated sustained anxiety reduction post-intervention, with participants in the music therapy groups reporting lower anxiety scores even weeks after the therapy. Moreover, satisfaction levels with the birth experience were higher in the music therapy groups, suggesting that this intervention not only reduces anxiety but also improves overall patient satisfaction with the childbirth process [19,20].

However, not all studies reported significant differences in anxiety reduction. For example, Drzymalski (2023) and Wu (2012) did not find substantial differences in anxiety scores between music therapy and control groups at all measured time points [21,45]. Also, Drzymalski's research on women with epidural catheters (2017) found that while music therapy was associated with higher anxiety scores immediately after placement, fewer women reported feeling "very much relaxed" compared to the control group one hour after the epidural technique [22]. Nevertheless, some studies reported no significant differences in anxiety reduction during cesarean sections despite the use of music therapy. For instance, Drzymalski (2020) illustrated that while preoperative music intervention led to slightly lower anxiety levels in the Mozart group compared to the control group, the differences in postoperative anxiety levels were not statistically significant [23]. Reza (2007) also found that intraoperative music delivered through headphones did not result in significant differences in postoperative anxiety scores, vomiting frequency, pain scores, or morphine requirements compared to the control group [37]. These discrepancies may be due to differences in study design, sample sizes, or the types of music used, highlighting the need for further research to identify the specific conditions under which music therapy is most effective. These findings suggest that incorporating music therapy into routine obstetric care could be a valuable strategy for reducing maternal anxiety. Given its non-invasive nature and positive impact on patient satisfaction, music therapy could be easily integrated into existing care protocols without significant additional costs. Moreover, the flexibility in delivery methods, as evidenced by the effectiveness of both conventional and virtual music therapy, allows for tailored interventions that meet the needs of diverse patient populations.

Physiological and Psychological Outcomes

Beyond subjective reports, several studies incorporated physiological measures to gauge the impact of music therapy. Ventura et al.'s (2012) exploration, focusing on cortisol levels and self-reported state anxiety, provides a refined understanding of the physiological changes induced by music interventions [44]. Additionally, the studies by Hepp et al. (2018) and Estrella-Juarez et al. (2023) shed light on the immediate and sustained effects on anxiety levels during and after medical procedures, emphasizing the role of music therapy in influencing both psychological and physiological domains [29,24].

Timing and Duration of Music Interventions

The temporal dimension of music interventions plays a pivotal role in their efficacy during the perinatal period. Drzymalski and Kakde both explored the impact of music on the patient's choice during the perioperative period, reporting reduced postoperative anxiety and lower pain scores [23,30]. These findings suggest that integrating music into the immediate preoperative period can positively influence emotional states and pain perception. Additionally, the duration of exposure to music interventions emerged as a variable of significance. Buglione's study, which allowed women to choose music to listen to during labor, reported decreased pain during the active phase of labor and reduced anxiety levels [16]. The flexible and adaptive nature of music interventions, ranging from brief exposures during medical procedures to more prolonged sessions during labor, highlights the versatility of this modality in catering to diverse clinical contexts. The temporal dynamics of music interventions should be considered in the design of future studies, with attention to optimal timing during perinatal care and the potential for sustained exposure for longer-lasting effects. Integrating music interventions into routine obstetric practices may require a nuanced understanding of the specific phases of pregnancy, labor, and postpartum recovery where music may exert the most significant impact on anxiety and pain.

Live Music Therapy

Furthermore, the incorporation of music therapy, as exemplified by a few studies, introduces live musical elements, indicating that the modality extends beyond passive listening. Garcia-Gonzalez's exploration of music therapy during pregnancy, using a CD of relaxing and instrumental music, introduces a live musical element to the therapeutic approach [25]. While this study demonstrated significantly lower scores in state and trait anxiety for the music therapy group, it also raises intriguing questions about the potential benefits of live music in perinatal care. Teckenberg-Jansson's study, which examined the emotional impact of preferred music choices on subjective well-being and stress markers, utilized live music therapy during a three-day intervention [42]. Although no significant group differences were noted in self-reported stress, the study provides valuable insights into the potential physiological effects of live music on stress responses, as evidenced by increased heart rate variability. The incorporation of live music therapy in obstetric settings introduces an interactive and dynamic element to the intervention, potentially fostering a more personalized and responsive therapeutic environment. Future research could delve deeper into the physiological mechanisms underlying the effects of live music, considering biomarkers such as cortisol levels, heart rate variability, and other stress-related indicators. Such investigations may unveil the full spectrum of benefits that live music therapy could offer in perinatal care.

Virtual Reality (VR) and Multisensory Approaches

Recent studies have ventured into the realm of virtual reality (VR) as a complementary tool in obstetric care. Almedhesh's investigation, incorporating VR with either music or Quranic verses during cesarean sections, expands the scope of non-pharmacological interventions [14]. The immersive experience provided by VR, coupled with auditory stimuli, creates a multisensory environment that has the potential to enhance maternal well-being. The integration of VR in obstetric settings offers a novel approach to managing anxiety and stress during procedures, going beyond the confines of traditional music interventions. The visual and auditory elements of VR may synergistically contribute to a more immersive and calming experience, potentially influencing both psychological and physiological responses. Further research is warranted to explore the specific mechanisms through which VR, coupled with music or other sensory stimuli, exerts its effects on perinatal well-being.

Variability in Outcomes

While most studies exhibit positive outcomes regarding anxiety reduction, an explorative interpretation is necessary. Drzymalski's study introduces a contrasting perspective, suggesting that music during epidural catheter placement may not necessarily improve pain or satisfaction and might elevate post-procedure anxiety [22]. These divergent findings prompt consideration of individual differences and contextual factors influencing the efficacy of music interventions.

Intrapartum Pain Management

Pain management during labor emerges as a critical facet of maternal care. Simavli’s comprehensive study emphasizes the effectiveness of music therapy in reducing pain perception throughout labor [39]. The study by Gokyildiz Surucu, focusing on active labor, further supports the idea that specific music interventions might alleviate perceived pain [26]. However, it is crucial to acknowledge that the impact of music therapy on pain can be multifaceted, as evidenced by the findings in the study conducted by Reza, where intraoperative music did not significantly alter postoperative pain [37].

Cultural Sensitivity and Preferences

One striking aspect that emerges from the reviewed studies is the influence of cultural preferences on the efficacy of music interventions. Hanprasertpong's study, which investigated the impact of Thai classical music during a medical procedure, revealed that music listening did not significantly affect post-procedure pain and anxiety [28]. This finding accentuates the importance of aligning musical choices with the cultural context in which interventions are implemented. Cultural sensitivity in designing music interventions is crucial for ensuring that the chosen music resonates with the preferences and expectations of the diverse population of perinatal individuals. The concept of 'music medicine' should extend beyond the universal appeal of certain genres and consider the rich tapestry of cultural expressions through music. Future studies should delve deeper into the relationship between culturally relevant music and therapeutic outcomes, potentially informing personalized approaches to music interventions based on cultural backgrounds.

Implications of the Results for Practice, Policy

The positive outcomes observed in anxiety reduction and pain management through music therapy interventions have significant implications for clinical practice. Integrating music therapy into routine maternal care, especially during critical interventions like cesarean sections, can contribute to a more positive birthing experience. Policymakers could consider incorporating music therapy as a non-pharmacological option within maternity care protocols. The collective evidence advocates for the incorporation of music therapy as an adjunctive non-pharmacological intervention. As these interventions are generally well-tolerated and present minimal risks, they offer a feasible and cost-effective option for enhancing the overall birthing experience. However, the heterogeneous nature of interventions and outcomes necessitates standardized guidelines for the implementation of music therapy in diverse clinical settings.

Limitations, and Future Research

Despite the compelling evidence supporting the efficacy of music therapy, it is essential to acknowledge the limitations inherent in the included studies. Variability in study designs, participant characteristics, and outcome measures may introduce heterogeneity, influencing the generalizability of the results. Additionally, the diversity in music therapy interventions, ranging from patient-selected music to virtual reality, introduces a challenge in drawing uniform conclusions. Some studies, such as Drzymalski's investigation, present contradictory outcomes, emphasizing the need for caution when generalizing the effectiveness of music therapy across all obstetric scenarios. The limited sample sizes in some studies, coupled with variations in the timing and duration of interventions, pose challenges in establishing definitive conclusions. Additionally, the subjective nature of self-reported outcomes, such as anxiety and pain scores, introduces a potential source of bias. Objective measures, including physiological markers and biomarkers associated with stress, could provide a more comprehensive understanding of the impact of music interventions on perinatal well-being. The exploration of individualized music interventions, considering the diverse preferences of perinatal individuals, represents a promising avenue for future research. Tailoring music interventions to individual preferences, cultural backgrounds, and specific phases of perinatal care could optimize therapeutic outcomes and contribute to a more personalized approach to obstetric care. Standardized protocols for music interventions, incorporating cultural relevance and individual preferences, could enhance the reproducibility and generalizability of findings. Future research should prioritize larger sample sizes and rigorous study designs to bolster the evidence base for the efficacy of music interventions in perinatal care. While this review aims to provide a comprehensive synthesis of existing evidence, it is vital to acknowledge potential limitations in the review processes employed. Inherent biases, such as publication bias favoring studies with positive outcomes, may influence the comprehensiveness of the review. Moreover, the exclusion of studies published in languages other than English might introduce a language bias. The search strategy, while rigorous, might have missed relevant studies due to variations in terminologies related to music therapy and maternal well-being. Rigorous systematic review methodologies, including comprehensive search strategies and transparent inclusion criteria, have been applied, but it is imperative to recognize and address these limitations. Future research should prioritize large-scale, well-controlled studies with standardized protocols to address existing gaps, ensuring robust evidence for guiding clinical practice and policy decisions. Additionally, investigating the long-term impacts of music therapy on maternal and neonatal outcomes will enrich our understanding of its sustained benefits.

## Conclusions

In conclusion, this systematic review navigates the intricate landscape of music interventions in perinatal care, highlighting their potential to alleviate anxiety and pain. The findings underscore the need for cultural sensitivity, recognizing the influence of individual preferences and cultural backgrounds on the efficacy of music interventions. The temporal dynamics of music interventions, whether integrated into the immediate preoperative period or during labor, present opportunities for optimizing their impact on emotional states and pain perception. The incorporation of live music therapy and virtual reality expands the scope of non-pharmacological interventions in obstetric care. Live music introduces an interactive and dynamic element, potentially modulating physiological responses to stress. Virtual reality, coupled with auditory stimuli, creates a multisensory environment that holds promise in enhancing maternal well-being during obstetric procedures. While the reviewed studies provide valuable insights, future research should address the limitations inherent in the current literature. Standardized protocols, larger sample sizes, and objective outcome measures are essential to advance our understanding of the therapeutic potential of music interventions in perinatal care. Integrating music into routine obstetric practices could usher in a harmonious and holistic approach to maternal well-being, contributing to a comprehensive model of perinatal care that embraces the therapeutic harmony of music.
